# Draft Genome Sequence of *Actinomyces glycerinitolerans* Strain G10^T^, Isolated from Sheep Rumen Fluid

**DOI:** 10.1128/genomeA.01589-16

**Published:** 2017-02-16

**Authors:** Susakul Palakawong Na Ayudthaya, Nikolaos Strepis, Peter Pristaš, Caroline M. Plugge

**Affiliations:** a Laboratory of Microbiology, Wageningen University, Wageningen, The Netherlands; b Laboratory of Systems and Synthetic Biology, Wageningen University, Wageningen, The Netherlands; c Institute of Biology and Ecology, Pavol Jozef Šafárik University, Košice, Slovak Republic; d Institute of Animal Physiology, Slovak Academy of Sciences, Košice, Slovak Republic

## Abstract

*Actinomyces glycerinitolerans* strain G10^T^, which was isolated from sheep rumen fluid, can metabolize a range of substrates, including complex carbohydrates to organic acids (OAs). Here, we report a 3.69-Mbp draft genome of *Actinomyces glycerinitolerans*.

## GENOME ANNOUNCEMENT

*Actinomyces glycerinitolerans* is a Gram-positive, pheomorhphic, nonmotile, anaerobic, and aerotolerant bacterium (1). Members of the genus *Actinomyces* have been found in natural environments (e.g., soil) or as commensals or pathogens of humans and other warm-blooded animals (2). Here, we report the draft genome sequence of a species belonging to the genus *Actinomyces* that originates from sheep rumen. *Actinomyces glycerinitolerans* (G10^T^) was found and isolated from a glycerol-tolerant enrichment of sheep rumen fluid (3).

Glycerol is an abundant and inexpensive carbon source that is generated as a by-product of biodiesel production (4). It has a high degree of reduction and therefore can enable increased yields of biofuels and reduced chemicals (3). Robust and strong organic acid-producing bacteria are interesting for biotechnological applications, such as glycerol-tolerant or -utilizing bacteria. On the other hand, glycerol is a generally recognized as safe animal food ingredient (3). *Actinomyces glycerinitolerans* can tolerate up to 25% glycerol and converts various complex substrates, including starch waste, to mainly succinate, lactate, and small amounts of acetate and formate (1).

Strain G10^T^ was cultured on a defined anaerobic bicarbonate-buffered medium with 500 mg/liter yeast extract and 20 mM glucose (1). Genomic DNA was extracted using the MasterPure complete DNA and RNA purification kit (Epicentre, Madison, WI). Sequencing was performed on an Illumina MiSeq sequencer with a read length of 250 bp and an insert size of 500 bp at GATC-Biotech, Konstanz, Germany. The genome size was first estimated by using KmerGenie (5) on the complete left side of the paired-end data. The quality of the reads was evaluated with FastQC (http://www.bioinformatics.babraham.ac.uk/projects/fastqc/). The assembly protocol (6) that was used included Ray-2.3.1 (7), with a k-mer value of 69. Annotation was carried out with an in-house pipeline, using Prodigal 2.5 (8) and InterProScan 5 (9). Mapping of the reads was conducted with Bowtie 2 (10).

The draft genome is 3.69 Mbp (99.99% of all filtered reads) and assembled into 19 scaffolds, with an *N*_50_ of 214,178 bp and a G+C content of 68.5%. The genome contains 58 tRNA genes, four 5S rRNA, one 23S rRNA, and one 16S rRNA. The genome is predicted to have 3,253 genes, of which 3,055 are protein encoding, and 1,464 were denoted as hypothetical.

Gene annotation with RAST (11) showed that the organism is capable of degrading carbohydrates (314 genes), including arabinose, fructose, galactose, glucose, mannose, maltose, lactose, trehalose, galacturonate, glucuronate, ribose, and xylose, as well as glycogen and maltodextrin. *A. glycerinitolerans* has potential resistance to osmotic stress (five genes) due to osmoregulation (aquaporin Z and outer membrane protein A precursor) and oxidative stress protection (eight genes), as it harbors genes like ferroxidase, zinc uptake regulation protein, organic hydroperoxide resistance, and superoxide dismutase. Self-defense mechanism clustered regularly interspaced short palindromic repeat (CRISPR) (CRISPR-associated protein [Cas] 1, 2, and 3) and Cas proteins were identified in *A. glycerinitolerans*.

### Accession number(s).

The *Actinomyces glycerinitolerans* strain G10^T^ whole-genome shotgun project has been deposited at DDBJ/EMBL/GenBank under the accession no. FQTT00000000. The version described in this paper is the first version.
